# Effects of manganese and zinc on the growth process of *Phytophthora nicotianae* and the possible inhibitory mechanisms

**DOI:** 10.7717/peerj.8613

**Published:** 2020-02-20

**Authors:** Yifang Luo, Aimei Yao, Mouyi Tan, Zhenlun Li, Ling Qing, Shuiying Yang

**Affiliations:** 1 Chongqing Key Laboratory of Plant Disease Biology, College of Plant Protection, Southwest University, Chongqing, China; 2 Chongqing Key Laboratory of Soil Multi-Scale Interfacial Process, College of Resources and Environment, Southwest University, Chongqing, China

**Keywords:** *Phytophthora nicotianae*, Manganese, Zinc, Gene expression, Antioxidant system, Inhibitory mechanisms

## Abstract

**Background:**

*Phytophthora nicotianae* is a fungal soil-borne pathogen that damages various plant species. Mancozeb and Zineb, fungicides containing manganese (Mn) and zinc (Zn) as the main components, are widely used to control the diseases caused by *Phytophthora*. However, the inhibition mechanism is still unclear. The purpose of this study was to examine the effects of Mn and Zn on *P. nicotianae* and to determine possible inhibitory mechanisms of Mn and Zn on sporangiogenesis of *P. nicotianae*.

**Methods:**

The mycelial growth, sporangium generation, zoosporogenesis and zoospore germination of *P. nicotianae* were observed under Mn and Zn treatments. The gene (*csn4* and *csn7*) expression levels of *P. nicotianae* in different growth stages were examined. *Csn4* and *csn7* gene expression, superoxide dismutase (SOD) activity, catalase (CAT) activity and malondialdehyde (MDA) content were tested at the stage of sporangiogenesis under different Mn and Zn concentrations.

**Results:**

Mycelial growth of *P. nicotianae* was significantly inhibited by Mn from ≥1 mg/L concentration and by Zn from ≥10 mg/L. The sporangia production, sporangia release, and zoospore germination of *P. nicotianae* were significantly reduced by Mn at all concentrations, while treatment with Zn from ≥0.5 mg/L concentration significantly inhibited the same processes. At the same concentration, the inhibition rate of Mn on the growth process of *P. nicotianae* was higher than that of Zn. The *csn4* and *csn7* gene transcription of *P. nicotianae* were significantly reduced by all treatments with Mn and Zn at the stage of sporangiogenesis. With the increase of Mn concentration, the activities of SOD and CAT increased to maxima and then decreased, and the content of MDA gradually increased during sporangiogenesis of *P. nicotianae*. The sporangia production of *P. nicotianae* was significantly positively correlated with the expression levels of the genes *csn4* and *csn7*.

**Conclusion:**

The inhibitory effect of Mn on the growth process of *P. nicotianae* was stronger than that of Zn, especially on sporangiogenesis and zoosporogenesis. A possible mechanism of the inhibitory effect on sporangiogenesis of *P. nicotianae* was that Mn and Zn acted by inhibiting the expression levels of the genes *csn4* and *csn7* and by affecting antioxidant enzyme activity (further resulting in lipid peroxidation) in the sporangium of *P. nicotianae*.

## Introduction

*Phytophthora nicotianae* is one of the most destructive soil-borne oomycetes pathogens in agriculture (Zhang et al., 2018; Lee et al., 2018). The fungus can infect tobacco, citrus, and other plants, causing serious economic losses (Jing et al., 2017). Through zoospores and oospores (Tan et al., 2018), pathogens infect the internal tissues of plants from roots, stems and leaves, thereby disrupting plant metabolism and leading to wilting, chlorisis and even death of the host plant (Gallup et al., 2018). Systemic diseases causing vascular bundle lesions in plants usually require the use of systemic fungicides for control (Han et al., 2019). Therefore, the selection of appropriate fungicides is of great significance for the control of diseases caused by *P. nicotianae*.

Manganese and zinc are essential microelements for plant growth and development. Nevertheless, research has demonstrated that both manganese and zinc can be used to control plant diseases. Mancozeb, manganese ethylene-bis-dithiocarbamate with zinc salts, is one of the most commonly used fungicides, and it can control plant diseases caused by *Phytophthora*, downy mildew, and other fungi (Panek & Tomsovsky, 2017; Herath et al., 2019). It is widely used in vegetables, fruit trees and field crops. The inhibition mechanism of mancozeb on the pathogens may involve reducing the activities of chitinase and β-1, 3-glucanase of fungal mycelia, causing the hydrolysis of glucan and chitin and leading to cell membrane disintegration, cytoplasm leakage and eventual cell death (Baider & Cohen, 2003; Yang & Zhang, 2012). Carmona et al. (2018) reported that manganese phosphite has significant control potential for diseases caused by *Phytophthora sojae*. In other research (Helfenstein et al., 2015; Li et al., 2016), zinc-containing agents could directly act on pathogens, initiate plant defenses, and reduce the virulence of pathogenic bacteria, thereby lessening bacterial plant damage. Savi et al. (2013) demonstrated that Zn compounds efficiently reduced fungal growth and fumonisin production, with the best results obtained from zinc perchlorate. At present, the inhibition mechanisms of manganese and zinc on sporangiogenesis of *P. nicotianae* are unclear.

The COP9 signaling complex (CSN), an important sensor of lipid oxidation involved in the cellular resistance to oxidative stress, plays a central role in regulating the post-translational processing of fungal proteins as well as in fungal development (Nahlik et al., 2010; Licursi et al., 2014; Pacurar et al., 2017). The fungal CSN is composed of eight subunits, *csn1-8* (Hu et al., 2007). Wang et al. (2010) have found that mycelial growth and the generation of conidia both declined in *csn1*, *csn4* and *csn7* mutants of *Neurospora crassa*. He et al. (2005) have reported that *csn2* mutation led to circadian rhythms of conidia becoming abnormal. Our previous research (Qiao et al., 2017) has indicated that the *csn4* gene is associated with the inhibitory effect of boron on sporangiogenesis of *P. nicotianae*. Except for the cited reports, there are few studies of CSN on the regulation mechanism of oomycete development. It is thus relevant to explore the role of CSN in the control of *P. nicotianae* infestation.

Antioxidant systems play an important role in microbial stress resistance. Microorganisms produce a small amount of reactive oxygen species (ROS) under stress, so superoxide dismutase (SOD), catalase (CAT) and other enzymes regulate ROS to restore the body’s oxidative level to normal and achieve the antioxidant effect (Jajda & Thakkar, 2012). However, if ROS are not cleared in time, the oxidative balance will be upset, and the plant tissue will be damaged. At that point, the malondialdehyde (MDA) content representing the degree of membrane lipid peroxidation is increased (Demirevska-Kepova et al., 2004). Therefore, the effects of Mn and Zn on *P. nicotianae* could be explored by analyzing SOD and CAT activities.

The purpose of this study was to compare the control effects of Mn and Zn on *P. nicotianae* by observing the mycelial growth, sporangia production and release, and zoospore germination under different treatments. The results showed that Mn had a greater inhibitory effect. The expression of *csn4* and *csn7* genes, the activity of SOD and CAT, and the content of MDA were detected to determine the effects of Mn and Zn on *P. nicotianae*. The possible inhibitory mechanism of Mn on *P. nicotianae* was explored, and the results provide a theoretical basis for the use of Mn against *P. nicotianae*.

## Materials and Methods

### Experimental materials and growth conditions of *P. nicotianae*

*Phytophthora nicotianae* was isolated from soils where tobacco black shank disease occurred as described previously (Qiao et al., 2017). Isolates were cultured in test tubes with oat medium and were stored at 15 °C. Pure isolates were later grown on oat medium for routine experiments.

The oat medium used for oomycete cultivation (per liter) contained 30 g oatmeal and 20 g agar (pH 7.5). The oat medium was autoclaved for 20 min at 115 °C.

The *csn4* (subunit 4) gene and *csn7* (subunit 7) gene (inserted into pMD19-T vectors) of *P. nicotianae* COP9 signaling complex were previously screened in our laboratory and stored at −80 °C in a 50% glycerol solution.

### Effect of Mn and Zn on mycelial growth of *P. nicotianae*

Mycelial growth of *P. nicotianae* was measured after inoculating in oat medium. A five mm diameter plug of mycelial agar was placed in the center of a petri dish containing oat medium in which 0 mg/L (control), 0.1, 1, 10, 100 and 200 mg/L Mn and 0 mg/L (control), 0.1, 0.5, 1, 5, 10 and 20 mg/L Zn had been prepared by adding Mn (NO_3_)_2_ and Zn (NO_3_)_2_, respectively, before pouring the plates. The medium was observed after incubating at 26 °C for 3 days. Results were reported as the diameter of the fungal colony in the petri dish minus the diameter of the agar plug (five mm). Each treatment contained three replicates.

### Effect of Mn and Zn on sporangium generation and zoosporogenesis of *P. nicotianae*

The mycelium was treated with MSS (fourmm MgSO4·7H2O, 50 mm KNO3, Fe-EDTA, pH 6.0) and incubated in the light at 26 °C for 12 and 24 h to initiate sporangiogenesis and zoosporogenesis of *P. nicotianae*, respectively. The effect of Mn and Zn on sporangiogenesis of *P. nicotianae* was assayed by supplying different concentrations of Mn and Zn in the above MSS solution until the ends of the mycelium had obviously expanded as observed under the microscope. The sporangia were counted by observation with a microscope using six fields. Released sporangia were expressed as a percentage based on the examination of 100 observed sporangia after being placed in 4 °C for 20 min and room temperature for 30 min. Each treatment contained three replicates.

### Effects of Mn and Zn on zoospore germination of *P. nicotianae*

The sporangia were treated with 1% KNO3 to induce rupture of the sporangia and the release of zoospores. The spore-containing medium was centrifuged at 4,000 r/min for 20 min; the supernatant was discarded, and sterile water was added. The centrifugation was repeated three times. The spores were resuspended in sterile deionized water at a concentration of 10^5^ cells/ml. Then, 0.1 ml spore resuspension was applied to WA medium (10 g agar per liter of water) containing different concentrations of Mn and Zn, and the suspension was cultured at 26 °C for 1 h in the dark and observed under a 40-fold objective. The length of germ tubes being more than half of the maximum diameter of the spore was set as the germination standard.

### Sample pre-preparation for the different stages of *P. nicotianae*

In order to detect gene expression during the different stages of *P. nicotianae*, nutrition stages and reproductive stages of *P. nicotianae* were prepared. The nutrition stage (mycelium) was obtained by incubating the fungal strain at 26 °C for 15 days; the reproductive stages containing sporangia were induced by treatment with MSS solution for 2, 6, 12 and 24 h. The zoospore release stage was induced by treatment for 48 h, and the culture was then kept at 4 °C for 20 min, after which the zoospore stage was prepared via holding at room temperature for 30 min followed by centrifugation; the zoospores were then collected.

In order to detect the effects of Mn and Zn on gene expression during the sporangiogenesis of *P. nicotianae*, mycelium at the sporangiogenesis stage was obtained by supplying different concentrations of Mn and Zn in the above MSS solution in the light for 12 h at 26 °C.

### Gene expression analysis by real-time quantitative PCR

Quantitative real time PCR (qRT-PCR) was performed to further verify the relationship between the *csn4* and *csn7* genes and the inhibitory effect of Mn on sporangiogenesis. Total RNA pools were extracted from the above preparations of *P. nicotianae* using TRIzol Reagent (Invitrogen, Shanghai, China) and were reverse transcribed using the PrimeScript™ RT reagent Kit with gDNA Eraser (Perfect Real Time) according to the manufacturer’s protocols. For PCR amplification, the following primers were used: Csn-FW TAGTCGTGTGTATAGCAGCATTTC and Csn-RV TCAAACTCCAGGAATCCCAAG for the subunit of *csn4*; CE-FW AACAAGCTCAGGAAACTGAC and CE-RV TGCCTTGAACTAATCCAGAG for the subunit of *csn7* and Tub-FW TTCATCGGTAACTCTACTGCC and Tub-RV ATCTCGTCCATACCCTCACC for beta tubulin. β-tubulin was used as an internal standard gene. The specificity of primers was checked by evaluation of melting curves and the presence of single amplicons as PCR reaction products. RT-PCR reactions were carried out in a total volume of 20 µL, each containing 1.6 µL of bacterium fluid (the target gene was inserted into a pMD19-T vector), 10 µL of the SYBR^®^ Premix Ex Taq™ II, 0.8 µL of each of the 10 µm primer solutions, and 6.8 µL of SDW. The qRT-PCR procedure was carried out with initial denaturation at 95 °C for 30 s followed by 40 cycles of 95 °C for 30 s, 58 °C for 30 s, 72 °C for 30 s, and one cycle at 95 °C for 15 s, and the melting curves were generated through increasing the temperature from 55 °C to 95 °C stepwise. The standard curves were established by using the target genes. The relative expression levels of *csn4* and *csn7* were calculated based on the ratios E*csn4*/E*tub* and E*csn7*/E*tub*, respectively.

### Transcription of *csn4* and *csn7* in different growth stages of *P. nicotianae*

Samples of 0.5 g of *P. nicotianae* mycelium under each treatment were scraped and ground with liquid nitrogen to obtain a fine powder, and the total RNA of *P. nicotianae* was reverse transcribed into cDNA by the Trizol method. Using cDNA as a template, the fluorescence quantitative standard curve established previously was used for the determinations. The relative expression levels of *csn4* and *csn7* were calculated according to the ratios E*csn4*/E*tub* and E*csn7*/E*tub*, respectively. The method of RNA extraction was the same for each developmental stage of *P. nicotianae*.

### Antioxidative enzyme extraction and activity assays

The fungal strain was grown on oat medium at 26 °C for 15 days followed by treatment with the MSS solutions containing different concentrations of Mn (0, 0.1, 1, 10, 100 and 200 mg/L) and Zn (0, 0.1, 0.5, 1, 5, 10 and 20 mg/L) in the light for 12 h at 26 °C until the ends of the mycelium had obviously expanded as observed under the microscope. Mycelia (0.3 g FW) were homogenized in 50 mm ice-cold phosphate buffer (pH 7.8) containing 0.2 mm EDTA-Na2 and 4% insoluble polyvinylpyrrolidone (one ml buffer/50 mg FW). The homogenate was centrifuged at 4 °C for 20 min at 12,000×*g*, and the supernatant was stored on ice. The activity of SOD was evaluated by the nitroblue tetrazolium photoreduction method, and the hydrogen peroxide decomposition method was employed for detection of CAT activity (Pignocchi et al., 2006).

### Evaluation of lipid peroxidation

The level of lipid peroxidation was described by the content of MDA in the mycelium. MDA was spectrophotometrically determined using the thiobarbituric acid (TBA) reactive substance assay according to Becana et al. (2012) with slight modifications. Mycelia (0.5 g FW) under different treatments were homogenized in eight ml of 0.1% trichloroacetic acid (TCA) and centrifuged at 4 °C for 15 min at 12,000*g* and then stored on ice. Two mm of the supernatant was mixed with 2.0 ml of 0.5% TBA in 10% TCA. The reaction mixture was heated for 20 min in a boiling water bath and quickly cooled. After centrifugation at 12,000*g* for 10 min, the absorbance of the clear supernatant was measured at 532 nm and 600 nm, and MDA content was calculated using 155 mM^−1^ · cm^−1^ as the molar absorbance coefficient. MDA levels were expressed as nmol per gram of wet mycelia.

### Statistical analysis

Statistical analysis and graphical work were carried out using Excel, SPSS Statistics, and Origin 9.0. The results were presented as means ± SD (standard deviation of means). Duncan’s multiple range test was used to test for significant differences. Values were considered to be statistically significant at *P* < 0.05. With concentration as the control and other indicators as variables, the correlations between the indicators were analyzed with the SPSS statistical software. All experiments were replicated three times.

## Results

### Effect of Mn and Zn on mycelial growth of *P. nicotianae*

The mycelial growth of *P. nicotianae* was inhibited by Mn and Zn (Table 1). Mycelial growth of *P. nicotianae* was significantly inhibited by Zn at concentrations ≥5 mg/L, while 0.1 mg/L Mn significantly inhibited the mycelial growth (*P* < 0.05). The colony diameters decreased as the concentrations of Mn and Zn increased. The inhibition rates of Mn and Zn at 0.1, 1 and 10 mg/L on the growth of *P. nicotianae* were 3.5%, 6.1%, 18.2% and 0%, 4.1%, 9.8%, respectively. The results suggested that the inhibitory effect of Mn on mycelial growth was stronger than that of Zn at the same concentration.

**Table 1:** Effects of Mn and Zn on mycelial growth, sporangia production, sporangia release and zoospore germination of *P. nicotianae*. The values are means ± SD of three replicates from repeating tests. Values in each column by different letters are significantly different at *P* < 0.05 (Duncan’s multiple range test).

Indexes	Manganese concentration (mg/L)						Zinc concentration (mg/L)						
	0	0.1	1	10	100	200	0	0.1	0.5	1	5	10	20
Colony diameter (mm)	67.7 ± 0.5a	65.4 ± 1.7b	63.6 ± 2.0b	55.4 ± 0.2c	53.5 ± 1.1c	45.6 ± 0.8d	67.7 ± 0.5a	67.8 ± 2.0a	66.1 ± 3.0ab	64.9 ± 2.0ab	64.0 ± 1.2bc	61.1 ± 1.7c	54.6 ± 2.3d
Sporangium number	75.2 ± 4.3a	51.2 ± 3.5b	29.5 ± 2.5c	21.2 ± 0.7d	16.1 ± 2.6e	9.6 ± 0.6f	75.2 ± 4.3a	60.0 ± 5.0b	58.7 ± 7.0b	44.3 ± 4.0c	42.7 ± 4.0c	40.0 ± 2.0cd	32.7 ± 2.1d
Sporangial release rate (%)	74.7 ± 4.2a	42.5 ± 2.7b	25.1 ± 1.6c	15.6 ± 0.7d	11.7 ± 1.6d	3.9 ± 0.3e	74.7 ± 4.2a	74.4 ± 2.6a	71.1 ± 2.4ab	68.8 ± 1.5b	67.3 ± 1.9bc	63.3 ± 3.0c	58.2 ± 3.2d
Zoospore germination rate (%)	72.9 ± 1.2a	58.1 ± 2.5b	53.8 ± 1.7c	51.1 ± 1.6c	41.8 ± 1.9d	37.0 ± 1.5e	72.9 ± 1.2a	69.7 ± 1.8b	58.6 ± 1.9c	60.7 ± 1.8c	51.6 ± 2.4d	43.4 ± 1.2e	44.7 ± 1.5e

### Effect of Mn and Zn on sporangiogenesis and zoosporogenesis of *P. nicotianae*

As shown in Table 1, both Mn and Zn inhibited the sporangiogenesis and zoosporogenesis of *P. nicotianae*. Treatment with Mn at all concentrations significantly inhibited sporangiogenesis and zoosporogenesis, with the inhibition rates ranging from 31.9% to 87.3% and 43.1% to 94.8%. The sporangiogenesis was also significantly inhibited by all concentrations Zn, with the inhibition rate ranging from 20.2% to 56.6%. Zn treatment significantly inhibited zoosporogenesis at concentrations ≥1 mg/L. The sporangial production and release rate per field were gradually decreased with the increase of Mn and Zn concentrations. The inhibition rates against sporangiogenesis of *P. nicotianae* were 31.9%, 60.8%, 71.8% and 20.2%, 41.1%, 46.8%, with Mn and Zn at 0.1, 1 and 10 mg/L, respectively. The inhibition rates on the release of *P. nicotianae* zoosporogenesis under 0.1, 1 and 10 mg/L Mn and Zn were 43.1%, 66.4%, 79.2% and 0.4%, 7.9%, 15.2%, respectively. The results indicated that at the same concentration, Mn had a stronger inhibitory effect on sporangium generation and zoosporogenesis than Zn.

### Effects of Mn and Zn on zoospore germination of *P. nicotianae*

Mn and Zn inhibited the germination of *P. nicotianae* spores (Table 1). The spore germination rate decreased with the increase in Mn and Zn concentrations. The spore germination rate was significantly reduced by Mn and Zn at all concentrations tested, with the respective amounts of control ranging from 20.3% to 49.3% and 4.4% to 38.7%. The inhibition rates of 0.1, 1 and 10 mg/L Mn and Zn on the germination of *P. nicotianae* were 20.3%, 26.2%, 29.9% and 4.4%, 16.7%, 40.4%, respectively. The results indicated that Mn had a stronger inhibitory effect on zoospore germination than Zn at the same concentration.

### *Csn4* and *csn7* genes expression at the different stages of *P. nicotianae*

**Figure 1:**
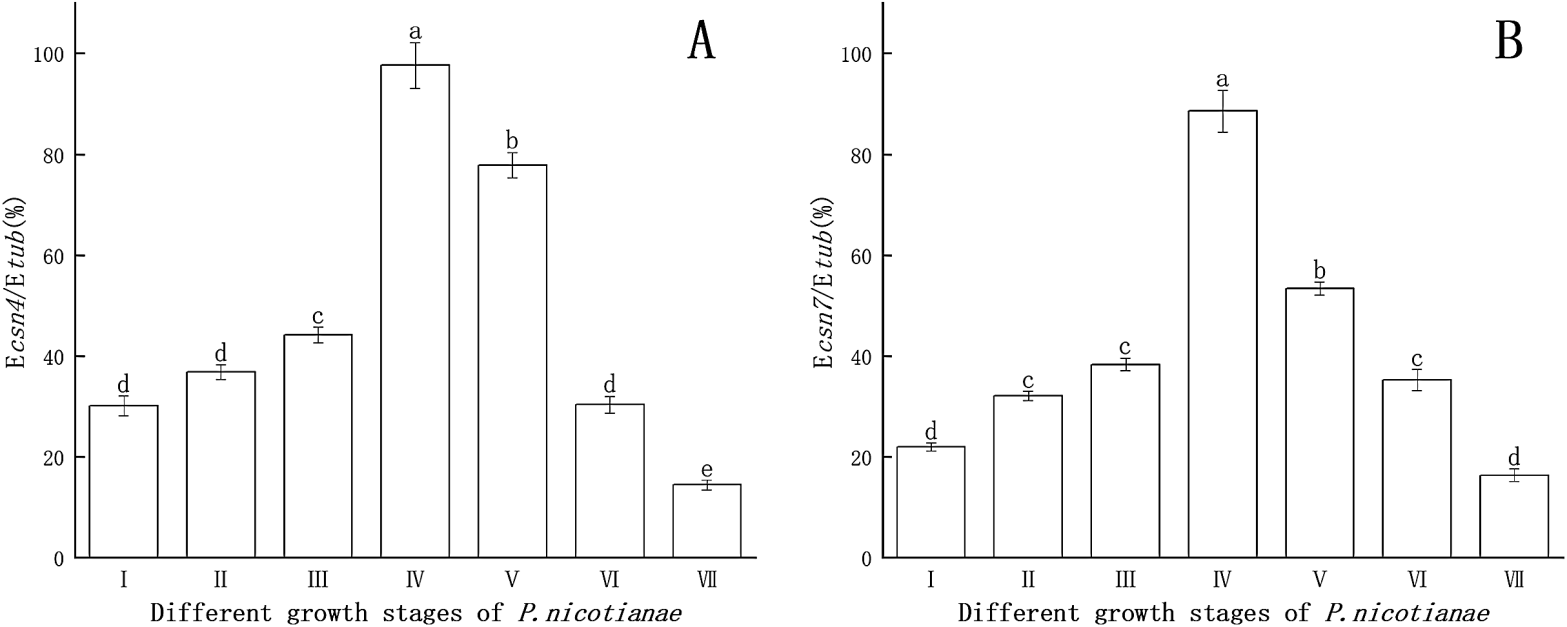
Gene expression of *csn4* and *csn7* at different stage of *P. nicotianae* based on E*csn4*/E*tub* and E*csn7*/E*tub*. (A) *Csn4* expression at different stage of *P. nicotianae*. (B) *Csn7* expression at different stage of *P. nicotianae*. Nutrition stage (I) mycelium was obtained by incubating strain at 26 °C for 15 days with oat medium. Reproductive stage (II, III, IV and V) mycelium was induced by MSS solutions (four mM MgSO4 · 7H_2_O, 50 mm KNO_3_, Fe-EDTA, pH 6.0) for 2, 6, 12 and 24 h after nutrition stage. Zoospore release stage (VI) mycelium was induced by MSS solutions for 48 h and next placed in 4 °C for 20 min. Zoospore stage (VII) room temperature for 30 min, centrifugation and collected zoospores after zoospore release stage. Different letters above columns are significantly different (*P* < 0.05) between experimental groups. Error bars are the standard error of the means (*n* = 3).

*Csn4* and *csn7* gene expression levels at different stages of *P. nicotianae* are shown in Fig. 1. *Csn4* and *csn7* expression levels were relatively higher at the sporangia production stage than at the zoospore stage. The reason for this was that *csn4* and *csn7* participate in the differentiation and synthesis of substances in the sporangium. When sporangia production was induced via MSS treatment for 12 h, the expression levels of both *csn4* and *csn7* reached their maximum (97.6% and 88.5%), indicating that this period is the peak and key period of mass synthesis of sporangium substances. Therefore, *P. nicotianae* treated with MSS for 12 h was used as the basis for determining the transcription levels of the *csn4* and *csn7* genes and for studying the relationship between the antioxidant system and the sporangium.

### Effect of Mn and Zn on gene expression in *P. nicotianae* at the stage of sporangiogenesis

The results of *csn4* and *csn7* gene expression at different stages of *P. nicotianae* were used as a reference. Gene expression analyses of *csn4* and *csn7* at different Mn or Zn concentrations were then tested individually for the effect on sporangiogenesis (induced for 12 h) of *P. nicotianae*.

**Figure 2:**
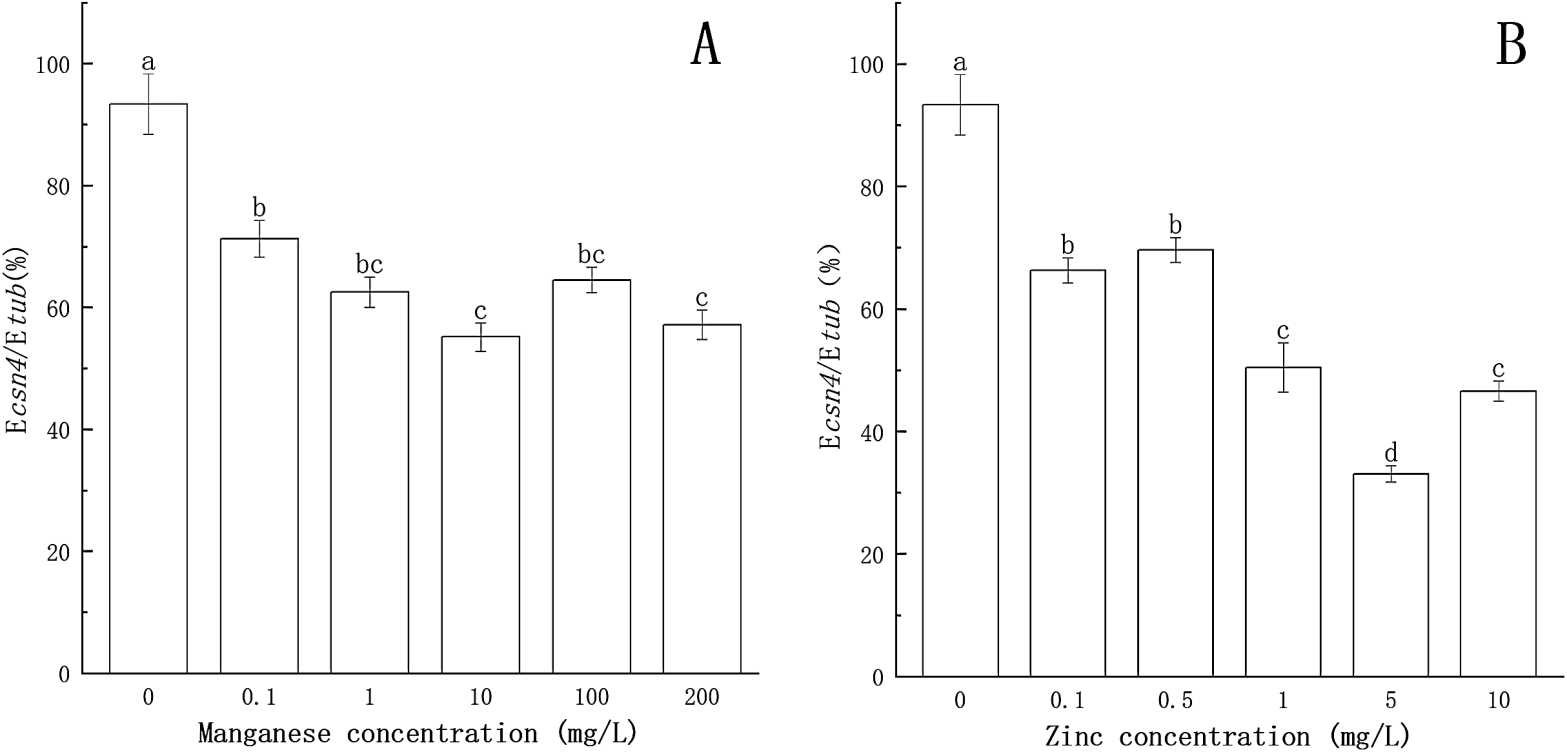
Effect of Mn and Zn on *csn4* expression of *P. nicotianae* at the stage of sporangiogenesis based on E*csn4*/E*tub*. (A) Effect of different Mn concentrations treatment on *csn4* expression of *P. nicotianae* at the stage of sporangiogenesis. (B) Effect of different Zn concentrations treatment on *csn4* expression of *P. nicotianae* at the stage of sporangiogenesis. Different letters above columns are significantly different (*P* < 0.05) between experimental groups. Error bars are the standard error of the means (*n* = 3).

The effects of Mn and Zn on the expression of *csn4* of *P. nicotianae* are shown in Fig. 2. When the concentration of Mn or Zn was 0, the transcription level of the *csn4* gene was the highest. Compared with the control, *csn4* expression levels were significantly suppressed (*P* < 0.05) by Mn and Zn at all concentrations, with the inhibition rates ranging from 23.7% to 40.9% and 25.4% to 64.6%. The transcription level of the *csn4* gene was the lowest when the concentration of Mn was 10 mg/L or when the concentration of Zn was 5 mg/L.

**Figure 3:**
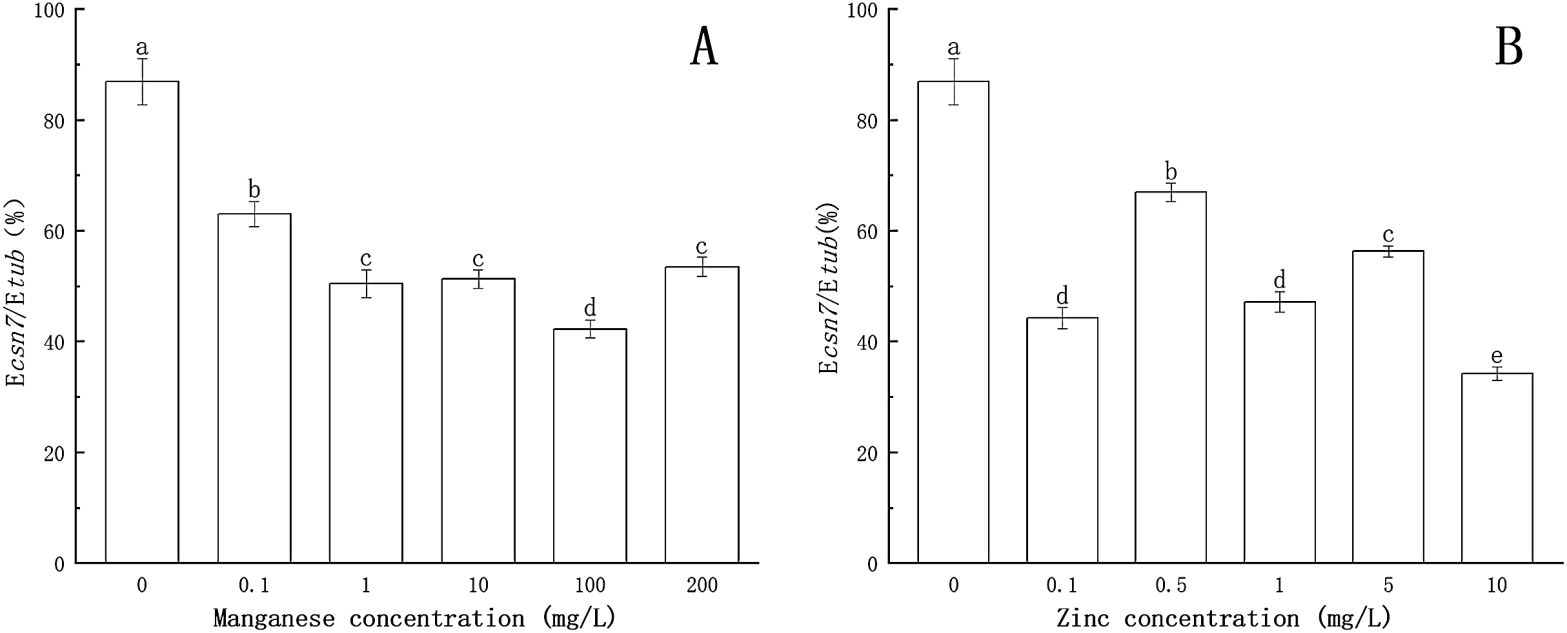
Effect of Mn and Zn on *csn7* expression of *P. nicotianae* at the stage of sporangiogenesis based on E*csn7*/E*tub*. (A) Effect of different Mn concentrations treatment on *csn7* expression of *P. nicotianae* at the stage of sporangiogenesis. (B) Effect of different Zn concentrations treatment on *csn7* expression of *P. nicotianae* at the stage of sporangiogenesis. Different letters above columns are significantly different (*P* < 0.05) between experimental groups. Error bars are the standard error of the means (*n* = 3).

As shown in Fig. 3, compared to the treatment without Mn or Zn, the inhibitory effects of Mn and Zn at different concentrations on the transcription level of the *csn7* gene of *P. nicotianae* were significant (*P* < 0.05). When the concentration of Mn or Zn was 0, the transcription level of the *csn7* gene was the highest. The inhibition of *csn7* transcription reached a maximum (51.4% and 60.6%) when the concentration of Mn was 100 mg/L or when the concentration of Zn was 10 mg/L.

These results clearly indicated that Mn and Zn had different effects on the expression of *csn4* and *csn7* in *P. nicotianae*.

### Effect of Mn-induced and Zn-induced oxidative stress on SOD activity in *P. nicotianae* at the stage of sporangia

**Figure 4:**
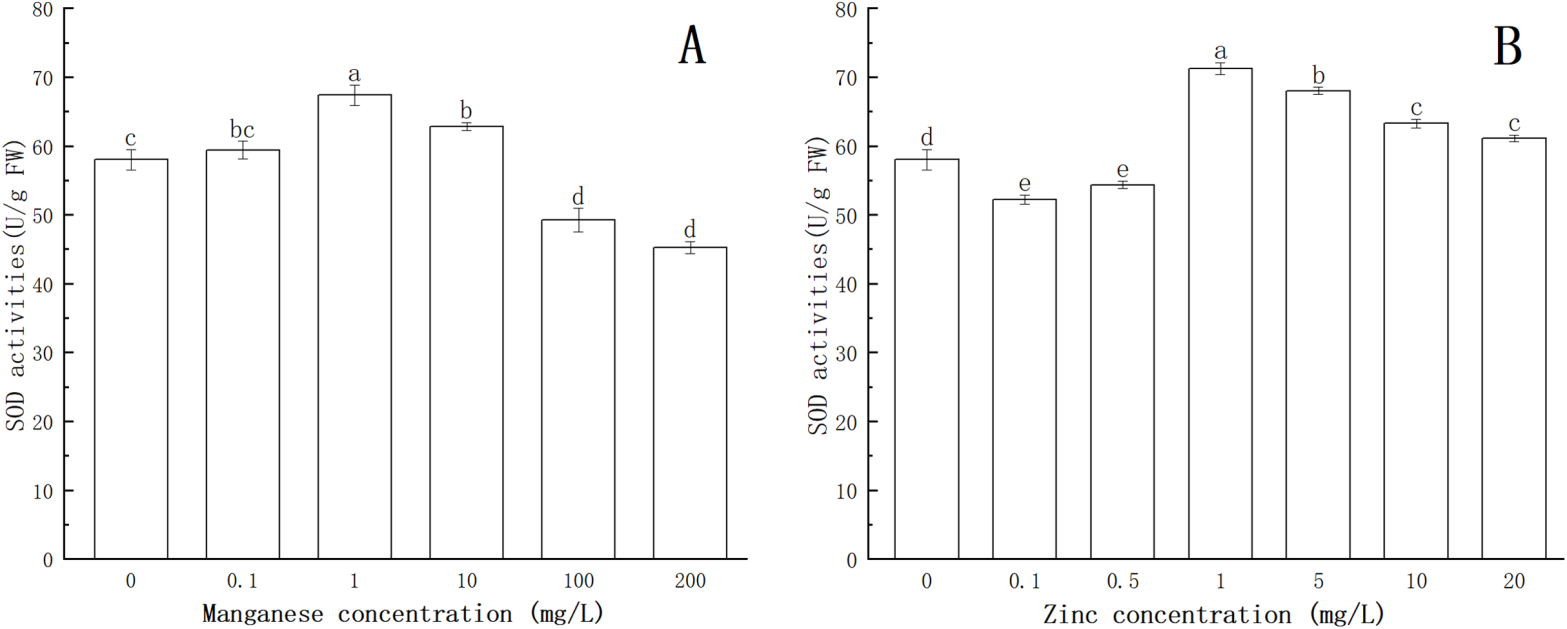
Effect of Mn and Zn induced oxidative stress on SOD activity of *P. nicotianae* at the stage of sporangiogenesis. (A) Effect of different Mn concentrations treatment on SOD activity at the stage of sporangiogenesis. (B) Effect of different Zn concentrations treatment on SOD activity at the stage of sporangiogenesis. Different letters above columns are significantly different (*P* < 0.05) between experimental groups. Error bars are the standard error of the means (*n* = 3).

As shown in Fig. 4, after being treated with Mn at a concentration of 0.1 mg/L, the activity of SOD was enhanced. When the concentration of Mn was 1 mg/L, the activity of SOD increased significantly (*P* < 0.05), reaching a maximum value (67.369 U/g FW). This indicated that with treatment by low concentrations of Mn, SOD could eliminate the excess ROS produced by *P. nicotianae* and thereby protect the plant. With the increase of Mn concentration (more than 1 mg/L ), the activity of SOD decreased gradually. SOD activity of *P. nicotianae* treated with Mn at 100 and 200 mg/L was significantly lower (*P* < 0.05) than that of the control group, indicating that the high concentrations of Mn were toxic to *P. nicotianae*, and the SOD produced was insufficient to remove the excess ROS.

The activity of SOD decreased significantly (*P* < 0.05) with 0.1 and 0.5 mg/L of Zn (Fig. 4). When the Zn concentration was 1 mg/L, SOD activity increased (*P* < 0.05) compared with the control group, and its maximum value was 71.23 U/g FW. With increasing Zn concentration, SOD activity gradually decreased, indicating that Zn concentration greater than 1 mg/L began to produce stress in *P. nicotianae*.

### Effect of Mn-induced and Zn-induced oxidative stress on CAT activity in *P. nicotianae* at the stage of sporangia

**Figure 5:**
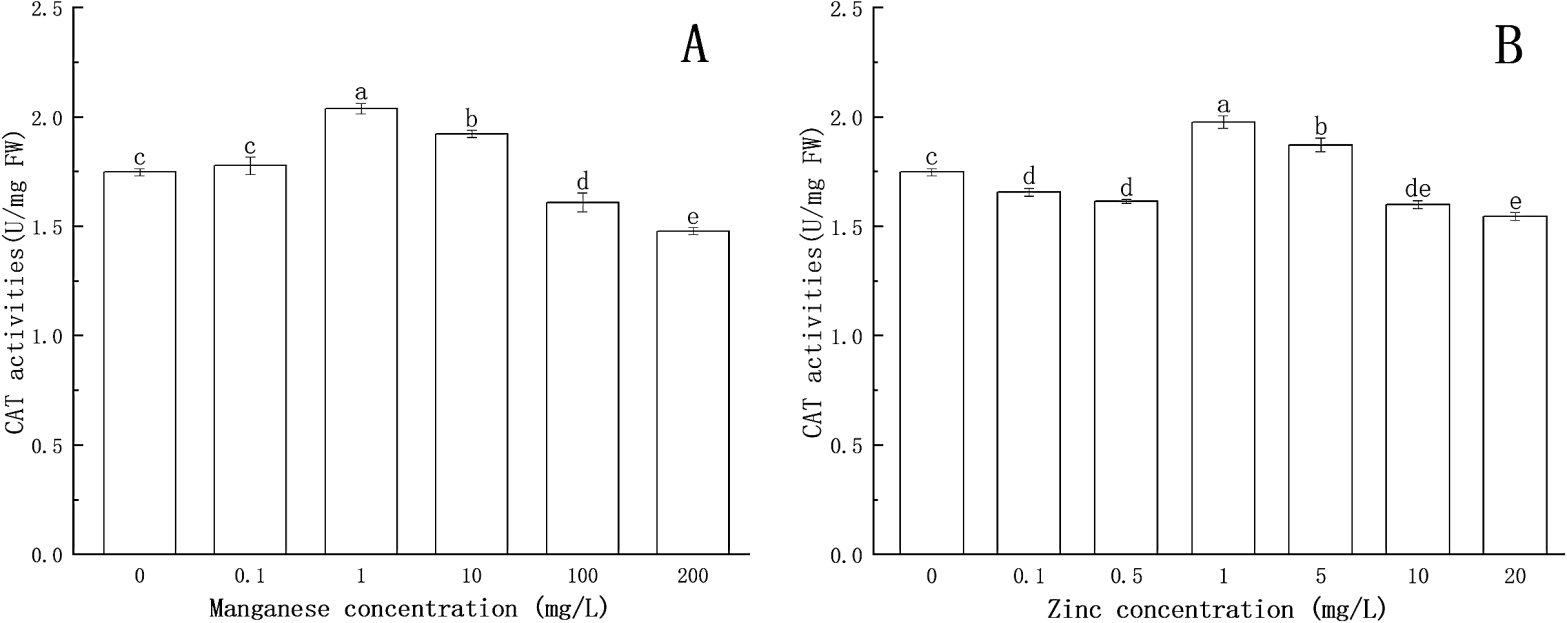
Effect of Mn and Zn induced oxidative stress on CAT activity of *P. nicotianae* at the stage of sporangiogenesis. (A) Effect of different Mn concentrations treatment on CAT activity at the stage of sporangiogenesis. (B) Effect of different Zn concentrations treatment on CAT activity at the stage of sporangiogenesis. Different letters above columns are significantly different (*P* < 0.05) between experimental groups. Error bars are the standard error of the means (*n* = 3).

The effects of Mn and Zn on CAT activity in *P. nicotianae* are shown in Fig. 5.

The CAT activity increased significantly (*P* < 0.05) at 1 mg/L of Mn, reaching a maximum value of 2.038 U/mg FW. The results showed that CAT could protect the mycelium under the treatment with low concentrations of Mn. The activity of CAT decreased gradually with the increase of Mn concentration above 1 mg/L. When the concentration of Mn was 100 mg/L or 200 mg/L, CAT activity was decreased significantly (*P* < 0.05) compared with the control group, indicating that high concentrations of Mn had a toxic effect on the normal development of the mycelium, and the toxic effect increased with increasing Mn concentration.

CAT activity was lower than that of the control group at 0.1 and 0.5 mg/L of Zn concentration. When Zn concentration increased to 1 mg/L, CAT activity increased significantly (*P* < 0.05) and reached a maximum value (1.976 U/mg FW), after which CAT activity gradually decreased. When Zn concentration was greater than 10 mg/L, CAT activity was significantly lower (*P* < 0.05) than that of the control group, indicating that Zn began to be toxic to the mycelium.

These results illustrated that Mn and Zn affected the balance of the antioxidant system in *P. nicotianae*. Similar findings have been reported in recent studies (Li et al., 2012; Canton et al., 2016).

### Effect of Mn and Zn on lipid peroxidation in *P. nicotianae* during the stage of sporangia

**Figure 6:**
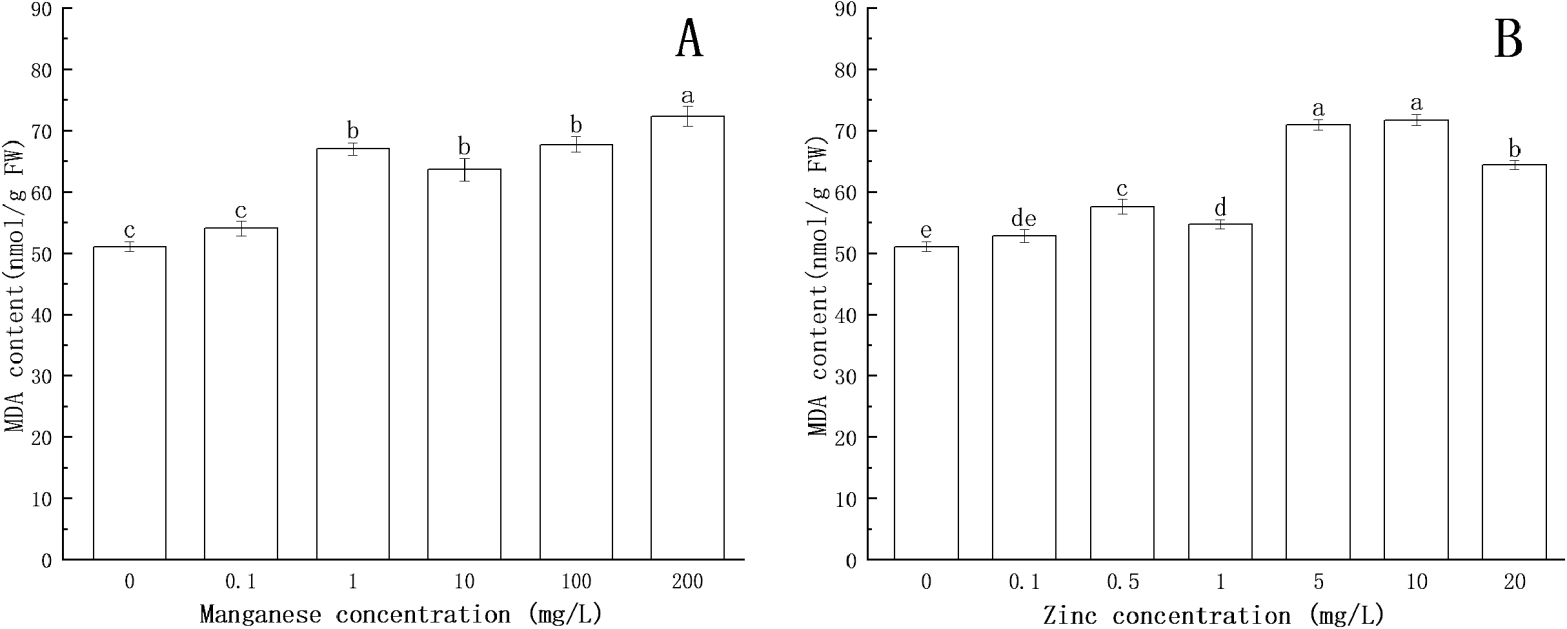
Effect of Mn and Zn lipid peroxidation based on MDA content of *P. nicotianae* at the stage of sporangiogenesis. (A) Effect of different Mn concentrations treatment on MDA content at the stage of sporangiogenesis. (B) Effect of different Zn concentrations treatment on MDA content at the stage of sporangiogenesis. Different letters above columns are significantly different (*P* < 0.05) between experimental groups. Error bars are the standard error of the means (*n* = 3).

Lipid peroxidation is an important indicator of oxidative stress. As one of the products of lipid peroxidation, the formation of MDA is deemed to be the most conclusive evidence of free radical-mediated reactions. As shown in Fig. 6, with the increase of Mn concentration, the MDA content increased. When the concentration of Mn was 1 mg/L, the MDA content increased significantly (*P* < 0.05), and the maximum MDA content (72.32 nmol/g FW) was obtained at 200 mg/L Mn.

Compared with the control group, the MDA content increased with increasing concentration of Zn. When the concentration of Zn was 0.5 mg/L, the content of MDA increased significantly (*P* < 0.05). When the concentration of Zn was 10 mg/L, the content of MDA reached the maximum (71.74 nmol/g FW).

All of the above results indicated that under the action of Mn and Zn, the accumulation of ROS in the somatic cells of *P. nicotianae* was in a state of oxidative stress, resulting in membrane lipid peroxidation. With the increase of ion concentration, the degree of membrane system damage increased, leading to the increases in MDA content. These results were consistent with the findings of Li et al. (2012).

### Correlation analysis

The correlation analysis of different indicators is shown in Tables 2 and 3. The sporangia production of *P. nicotianae* was significantly positively correlated with the expression levels of *csn4* and *csn7* under Mn and Zn treatment. This showed that the number of sporangia decreased with the decrease in gene expression. There were extremely significant positive correlations between SOD activity, CAT activity and MDA content under Mn treatment. These results indicated that *csn4* and *csn7* of *P. nicotianae* are related to sporangium production. Mn and Zn might inhibit sporangium generation by inhibiting the expression levels of *csn4* and *csn7* to control the reproduction of *P. nicotianae*. Also, Mn could significantly affect the activity of enzymes to inhibit *P. nicotianae* during the stage of sporangia.

**Table 2:** Correlation analysis of sporangium generation, the expression levels of gene(*csn4* and *csn7*), SOD activity, CAT activity and MDA content of *P. nicotianae* under Mn treatment. With Mn concentration (three replicates) as the control and other indicators as variables, the correlations between the indicators were analyzed with the SPSS statistical software.

Correlations	Sporangium number	E*csn4*/E*tub* (%)	E*csn7*/E*tub* (%)	SOD activity (U/g FW)	CAT activity (U/mg FW)	MDA content (nmol/g FW)
Sporangium number	1					
E*csn4*/E*tub* (%)	0.914**	1				
E*csn7*/E*tub* (%)	0.924**	0.817**	1			
SOD activity (U/g FW)	−0.562*	−0.541*	−0.417	1		
CAT activity (U/mg FW)	−0.682**	−0.663**	−0.559*	0.712**	1	
MDA content (nmol/g FW)	−0.848**	−0.715**	−0.804**	0.658**	0.801**	1

**Notes:**

* Significantly correlated (*P* < 0.05).

** Extremely significant correlation (*P* < 0.01).

**Table 3:** Correlation analysis of sporangium generation, the expression levels of gene(*csn4* and *csn7*), SOD activity, CAT activity and MDA content of *P. nicotianae* under Zn treatment. With Zn concentration (three replicates) as the control and other indicators as variables, the correlations between the indicators were analyzed with the SPSS statistical software.

Correlations	Sporangium number	E*csn4*/E*tub* (%)	E*csn7*/E*tub* (%)	SOD activity (U/g FW)	CAT activity (U/mg FW)	MDA content (nmol/g FW)
Sporangium number	1					
E*csn4*/E*tub* (%)	0.824**	1				
E*csn7*/E*tub* (%)	0.662**	0.539*	1			
SOD activity (U/g FW)	−0.578*	−0.540*	−0.084	1		
CAT activity (U/mg FW)	−0.505*	−0.585*	−0.083	0.993**	1	
MDA content (nmol/g FW)	−0.392	−0.560*	0.111	0.226	0.226	1

**Notes:**

* Significantly correlated (*P* < 0.05).

** Extremely significant correlation (*P* < 0.01).

## Discussion

### Effects of Mn and Zn on the Growth of *P. nicotianae*

*Phytophthora nicotianae* is an important pathogenic microorganism with a wide range of hosts; the fungus causes root and stem diseases of various crops (Bittner & Mila, 2016). Previous research (Baider & Cohen, 2003; Helfenstein et al., 2015; Shi et al., 2006) reported positive effects of manganese-containing fungicides and zinc-containing agents on fungal diseases. Savi et al. (2013) showed that zinc compounds significantly inhibited *Fusarium verticillioides* growth from ≥25 nmol/L concentration, and all treatments inhibited conidia production. Manganese-containing fungicides such as mancozeb could inhibit the growth of *P. nicotianae*, and the highest inhibition rate was 62.7% (Panek & Tomsovsky, 2017). This study clearly validated the inhibitory activities of Mn and Zn against *P. nicotianae*. The inhibition of Mn and Zn on the growth of *P. nicotianae* increased with the increase of concentrations. The inhibitory effects of Mn on mycelial growth, sporangium generation, zoosporogenesis and zoospore germination of *P. nicotianae* were stronger than those of Zn.

### Effect of Mn and Zn on csn4 and csn7 gene expression in *P. nicotianae* at the stage of sporangiogenesis

COP9 signalosome (CSN) has profound effects on the regulation of the ubiquitin proteasome system, mycelial growth and conidial yield (Pacurar et al., 2017; Licursi et al., 2014; Hind et al., 2011). The filamentous fungus *Aspergillus nidulans* survives without CSN but is unable to complete sexual development (Nahlik et al., 2010). Zhou et al. (2012) used mutation analysis to confirm that the JAMM domain of the CSN-5 subunit is responsible for NEDD8 cleavage from cullin proteins in *N. crassa* and found that CSN integrity plays major roles in hyphal growth, conidial development, and circadian function in *N. crassa*. He et al. (2005) suggested that CSN mutation leads to abnormal circadian rhythms of conidia. The present study indicated that Mn and Zn significantly inhibited the expression of *csn4* and *csn7* during sporangiogenesis of *P. nicotianae*. The sporangium generation of *P. nicotianae* was significantly positively correlated with *csn4* and *csn7* expression levels. All results indicated that the *csn4* and *csn7* genes had a great impact on sporangia production of *P. nicotianae*. In addition, Nahlik et al. (2010) believed that CSN is necessary to handle oxidative stress, as it appears to protect the fungus during the internal ROS signaling events triggering development. The induction of antioxidant enzyme activity and membrane lipid peroxidation was also observed in *P. nicotianae* treated with Mn and Zn. However, the correlation between destruction of the antioxidant system and *csn4* and *csn7* gene expression needs further exploration.

### Effects of Mn and Zn on the anti-oxidation system in the sporangium of *P. nicotianae*

Previous researchers have reported that catalase and glutathione transferase were both activated when the fungus *Pisolithus tinctorius* was exposed to Mn (Canton et al., 2016); SOD and CAT activities in filamentous fungus *Penicillium janthinellum* also increased when treated with Mn (Ling et al., 2017). In this research, the CAT and SOD activity first increased with the increase in Mn concentration and then decreased after reaching the maximum values, indicating that *P. nicotianae* could achieve a certain balance between the production and clearance of ROS by increasing the activity of SOD and CAT to protect itself under low concentrations of Mn. High concentrations of Mn are toxic to *P. nicotianae* due to membrane lipid peroxidation from the imbalance in the antioxidant system. These results were consistent with the findings of Li et al. (2012), Canton et al. (2016) and Li et al. (2012). With increasing Zn concentration, CAT activity and SOD activity decreased slightly, then reached their maximum values and finally decreased. The cause of this phenomenon remains unclear and needs further study. In conclusion, possible reasons for the ability of Mn to control sporangium generation of *P. nicotianae* were the destruction of antioxidant levels and the production of membrane lipid peroxidation.

## Conclusion

The inhibitory effect of Mn on the growth process of *P. nicotianae* was stronger than that of Zn, especially on sporangiogenesis and zoosporogenesis. The possible inhibitory mechanism on sporangiogenesis of *P. nicotianae* involved Mn and Zn inhibiting sporangia production by reducing the expression levels of the genes *csn4* and *csn7* and affecting the antioxidant enzyme activity (further resulting in lipid peroxidation) in the sporangia of *P. nicotianae*.

## Supplemental Information

10.7717/peerj.8613/supp-1 Supplemental Information 1 Raw data on the effects of Mn on mycelial growth, sporangia production, sporangia release, zoospore germination, *csn4* and *csn7* expression, SOD activity, CAT activity, and MDA content of *P. nicotianae*. All experiments were replicated three times.

10.7717/peerj.8613/supp-2 Supplemental Information 2 Raw data on the effects of Zn on mycelial growth, sporangia production, sporangia release, zoospore germination, *csn4* and *csn7* expression, SOD activity, CAT activity and MDA content of *P. nicotianae*. All experiments were replicated three times.

10.7717/peerj.8613/supp-3 Supplemental Information 3 Raw data of *csn4* and *csn7* expression at different stage of *P. nicotianae* based on E*csn4*/E*tub* and E*csn7*/E*tub*. All experiments were replicated three times.

## Additional Information and Declarations

### Data Availability

The following information was supplied regarding data availability: The raw measurements are available in the Supplemental Files.
